# Appendiceal adenocarcinoma found by surgery for acute appendicitis is associated with older age

**DOI:** 10.1186/s12893-021-01224-0

**Published:** 2021-05-02

**Authors:** John P. Skendelas, Victor S. Alemany, Vincent Au, Devika Rao, John McNelis, Peter K. Kim

**Affiliations:** 1 Jacobi Medical Center, Bronx, NY USA; 2 North Central Bronx Hospital, Bronx, NY USA; 3 Montefiore Medical Center, Bronx, NY USA; 4 Albert Einstein College of Medicine, Bronx, NY USA; 5 Jacobi Medical Center, 1400 Pelham Parkway, Building 1, Room 510, Bronx, NY 10461 USA

**Keywords:** Acute appendicitis, Adenocarcinoma, Neuroendocrine tumor, Mucinous neoplasm, Emergency general surgery, Acute care surgery

## Abstract

**Background:**

Appendectomy for acute appendicitis is the most common procedure performed emergently by general surgeons in the United States. The current management of acute appendicitis is increasingly controversial as non-operative management gains favor. Although rare, appendiceal neoplasms are often found as an incidental finding in the setting of appendectomy. Criteria and screening for appendiceal neoplasms are not standardized among surgical societies.

**Methods:**

The National Surgical Quality Improvement Program (NSQIP) database was queried for all patients who underwent appendectomy over a 9-year period (2010–2018). Over the same time period, patients who underwent appendectomy in two municipal hospitals in The Bronx, New York City, USA were reviewed.

**Results:**

We found a 1.7% incidence of appendiceal neoplasms locally and a 0.53% incidence of appendiceal tumors in a national population sample. Both groups demonstrated an increased incidence of appendiceal carcinoma by age. This finding was most pronounced after the age of 40 in both local and national populations. In our study, the incidence of appendiceal tumors increased with each decade interval up to the age of 80 and peaked at 2.1% in patients between 70 and 79 years.

**Conclusions:**

Appendiceal adenocarcinomas were identified in patients with acute appendicitis that seem to be associated with increasing age. The presence of an appendiceal malignancy should be considered in the management of older patients with acute appendicitis before a decision to embark on non-operative therapy.

## Background

The incidence of appendicitis in the United States has been reported between 82 and 111 per 100,000 population per year, with a life-time risk of 1 in 15 (6.7%) [1]. Appendectomy for acute appendicitis is the most common emergency intra-abdominal operation performed by general surgeons, and approximately 300,000 appendectomies are performed annually in the USA alone [2]. In recent years, there has been increased interest in non-operative management of acute appendicitis as safe and feasible first-line therapy [3], similar to the management approach in other countries [4–7]. Historically, antibiotic therapy has been used in 40–45% of patients in Europe compared to fewer than 5% in the United States [8, 9]; however, this approach has been challenged due to the ongoing “Comparison of Drugs versus Appendectomy” (CODA) trial, which found that early outcomes of medical management of appendicitis, with or without appendicolith, was non-inferior to surgery [10].

The COVID-19 pandemic has had extensive effects across the healthcare spectrum, including the management of acute appendicitis. Conservative management of mild appendicitis was practiced in several centers across the globe during the pandemic [11]. Non-operative management during the COVID-19 pandemic did not increase complications in one center [12], although several questions remain regarding long-term outcomes in these patients. Unintended consequences of non-operative management may include increased incidence of antibiotic-resistant organisms and alterations to the microbiome [3]. Nevertheless, this approach has been adopted by the American College of Surgeons during the current pandemic and will likely continue [13].

This shift in clinical practice has raised concerns regarding the risk of missing an appendiceal neoplasms in patients who receive non-operative therapy [14]. In the CODA trial, neoplasms were identified in 9 participants (0.6%), in spite of strict inclusion criteria to screen for underlying or likely malignancy [10]. Similarly, in the post-hoc analysis of the “Multicenter Study of the Treatment of Appendicitis in America: Acute, Perforated, and Gangrenous” (MUSTANG) trial [15, 16], neoplasms were associated with patient age greater than 40 and appendiceal size greater than 1 cm on cross-sectional imaging.

While the overall incidence remains low, there are neither established criteria to determine which patients are eligible for medical management nor criteria for patients who may require oncologic surveillance after non-operative treatment for appendicitis. The aims of this study were (1) to determine the incidence of appendiceal neoplasms from the National Surgical Quality Improvement Program (NSQIP) database and (2) compare these findings in two urban municipal hospitals in the Bronx, New York City.

## Methods

The National Surgical Quality Improvement Program (NSQIP) personal use file database was queried using the ICD-9 codes from May 1, 2010 to December 31, 2018 for ‘appendicitis”: 540 (540.0, 540.1, 540.9), 541, 542; “appendectomy as an outpatient procedure”: 44,950, 44,955, 44,960, 44,870, 44,979, 44,900, 44,901; “appendectomy as an inpatient procedure”: 45.72, 45.73, 47, 47.01, 47.09, 47.1, 47.11, 47.19, 47.2, 47.92, 47.99; “neoplasm of the appendix”: 153.9 and 152.9; and the ICD 10 codes for “appendicitis” K35, K36, “other appendicitis” K37 and K38; “neoplasm of the appendix”, “neoplasm of the intestinal tract”, “small intestine and colon unspecified” C17.9, C18.9, C26.0 and C18.1 and for “appendectomy”: 0DTJ0ZZ, 0DTJ4ZZ, 0DTJ7ZZ, 0DTJ8ZZ. During this time period there were over 2.8 million patients in the database. Cases of appendiceal neoplasm were then categorized by age, type of procedure, and pathologic diagnosis. In the database there were only three pathologies of appendiceal neoplasm specified: adenocarcinoma, malignant carcinoid, and unspecified. Cases with known benign pathology after appendectomy were excluded from analysis.

Institutional data were retrospectively collected from the electronic medical records of all patients diagnosed with acute appendicitis in the period January, 2010 through December, 2018 in two Bronx municipal hospitals. Records were further confirmed with an institutional *Tumor Registry and Pathology* database to ensure inclusion of all malignant cases. All 1989 surgical pathology reports were reviewed to evaluate for neoplasms in appendectomy specimens. Data acquired included age at diagnosis, sex, prior colonoscopy records, radiographic findings, as well as final pathology and oncologic outcomes.

Continuous variables were expressed as medians with interquartile range (IQR). Categorical variables were described with number counts and percentages. Continuous variables were compared using Mann–Whitney U test. Categorical variables were compared using Chi-square tests. All statistical tests were two-tailed and p-value less than 0.05 was considered significant. All analyses were performed using SPSS Version 27.0 (IBM Corp, Armonk, NY, USA). All data collection, chart review, and methods were performed with approval by the hospital’s Institutional Review Board (IRB) of the Albert Einstein College of Medicine (IRB Protocol #2017-7530) and in accordance with all relevant guidelines and regulations.

## Results

**Fig. 1:**
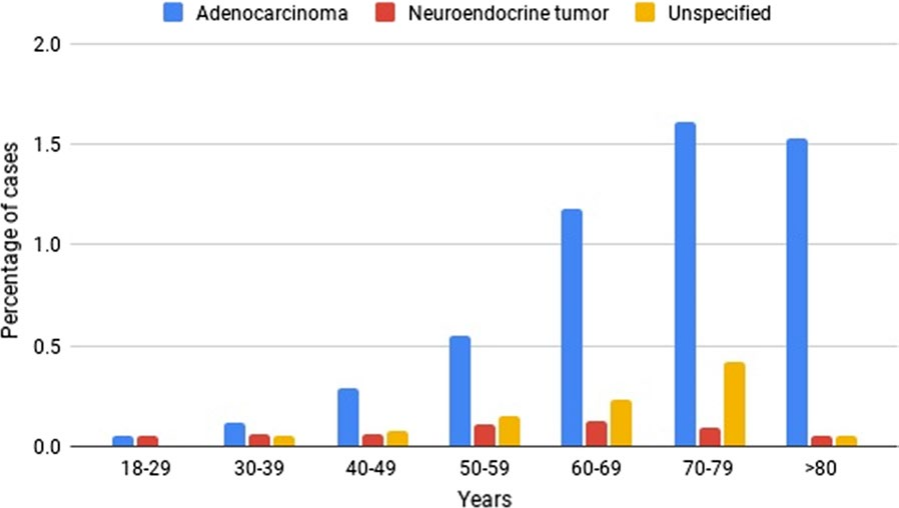
The incidence of adenocarcinoma, neuroendocrine, and low grade appendiceal mucinous neoplasm (LAMN) obtained from the National Surgical Quality Improvement Program (NSQIP) database from 2010 to 2018 categorized into 10 year age groups

The National Surgical Quality Improvement Program (NSQIP) database was queried between 2010 and 2018. A total of 154,596 appendectomies performed in adult (age ≥ 18 years) patients were identified, which included 812 cases of appendiceal cancer (for an incidence of 0.53%). The incidence of appendiceal tumors stratified by age are summarized in Fig. 1. The combined incidence of appendiceal neoplasms identified in these data are 0.4%, 0.8%, 1.6%, 2.1%, and 1.6% respectively for age 40–49, 50–59, 60–69, 70–79, and > 80. For patients older than 40 years, 1% of appendectomy specimens demonstrated an appendiceal neoplasm. From the 812 appendiceal neoplasms, 681 adenocarcinomas of the appendix (0.4%) were identified. For these patients, 290 underwent appendectomy and 524 underwent colectomy; 133 patients underwent both procedures.

**Fig. 2:**
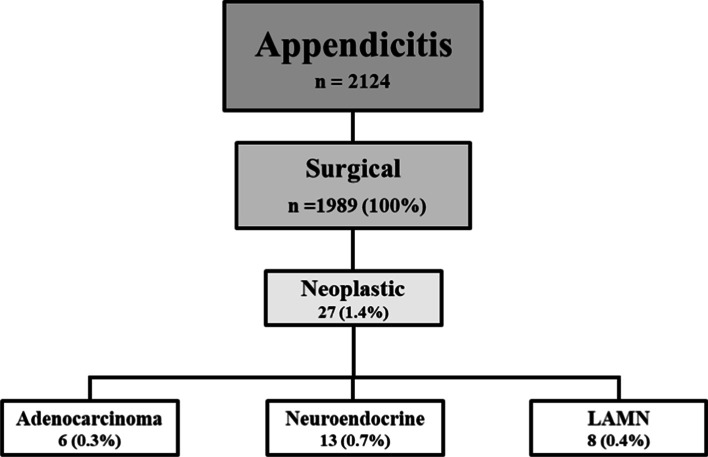
The total number of local cases of appendicitis and those managed surgically reviewed from two Bronx municipal hospitals for the period 2010 through 2018. The number of cases found to be appendiceal cancers are further categorized into adenocarcinoma, neuroendocrine, and low grade appendiceal mucinous neoplasm (LAMN)

A total of 2124 medical charts from patients diagnosed with acute appendicitis were reviewed in two municipal hospitals using the electronic medical records from 2010 through 2018. At these institutions, 1989 patients (93.6%) underwent operative treatment for acute appendicitis, with 1104 patients (52.0% of total surgeries) undergoing laparoscopic appendectomy, 852 for open surgery (40.1%) and 32 (1.5%) who underwent conversion from laparoscopic to open approach. From the operative cohort, 27 patients were diagnosed with appendiceal neoplasm after the pathologic study of the removed appendix which reflected an incidence of 1.4% in this study population over 9 years (Fig. 2).

The median age of the neoplasm cohort was 49.0 years IQR [39.3–54.2]), with approximately 2:1, female to male ratio (70.4% female). Remaining demographics and clinical factors are presented by non-adenocarcinoma (low-grade appendiceal mucinous neoplasm or LAMN, neuroendocrine) and appendiceal adenocarcinoma in Table 1. Of note, 11 of 27 patients (40.7%) presented with complicated disease (e.g. perforation, small bowel obstruction, or malignant ascites), whereas only 7 of 27 patients (25.9%) had a prior colonoscopy. In addition, all neuroendocrine tumors were Grade 1 and categorized as benign.

**Table 1:** Demographics

	Appendiceal adenocarcinoma (n = 6)	Composite non-adenocarcinoma^a^ (n = 21)	p-value
Age—years [IQR]	65.0 [52.8–75.3]	47.0 [25.5–54.5]	0.004
Female sex—no. (%)	5 (83)	14 (67)	0.633
Previous colonoscopy—no. (%)	2 (33)	5 (24)	0.633
Imaging size, median—cm [IQR]	1.4 [1.3–3.0]	1.2 [1.1–1.8]	0.263
Emergency department presentation—no. (%)	6 (100)	18 (86)	0.810
Operative intervention—no. (%)			0.552
Open	4 (67)	9 (43)	
Laparoscopic	2 (33)	11 (52)	
Converted	0	1 (5)	
Complicated—no. (%)^b^	3 (50)	8 (38)	0.662

*IQR* interquartile range

^a^Patients with appendectomy specimen pathology consistent with low-grade appendiceal mucinous neoplasm or neuroendocrine tumors

^b^Complicated cases included radiologic evidence of perforation (n = 7), small bowel obstruction (n = 3), and malignant ascites (n = 1)

The entire series of patients with appendiceal neoplasms are presented in Table 2 with associated age at diagnosis, appendiceal size on imaging, surgical approach, pathology, and stage. The final pathology revealed six cases of adenocarcinoma (0.3% of appendectomy specimens), 13 neuroendocrine tumors (0.7%), and eight cases of low-grade appendiceal mucinous neoplasms (LAMN, 0.4%). Twenty (74.1%) of the patients were 40 years or older at the time of diagnosis. Moreover, the diameter of the appendix was greater or equal to 1.0 cm on pre-operative imaging in 25 of 27 (92.6%) patients with appendiceal cancer. All patients with LAMN (n = 8) had an appendiceal diameter greater than 1.0 cm with imaging findings consistent with mucocele.

**Table 2:** Summary of appendiceal neoplasms

Patient ID	Age at presentation (years)	Appendiceal imaging size (cm)	Pathology	Stage (TNM)	Surgical procedure
1	73	3.1	Adenocarcinoma	IIA (T3, N0, M0)	Open right hemicolectomy
2	67	3.0	Adenocarcinoma	IV	Diagnostic laparoscopy with appendectomy, omental and peritoneal biopsy
3	63	1.2	Adenocarcinoma	IIA (T3, N0, M0)	Open right hemicolectomy
4	82	1.4	Adenocarcinoma	IV (T4, N2, M1)	Open right hemicolectomy
5	54	1.4	Adenocarcinoma	IIA (T3, N0, M0)	Laparoscopic appendectomy with interval open right hemicolectomy
6	49	1.3	Adenocarcinoma	IV (T3, N2, M1)	Open right hemicolectomy
7	23	1.3	Neuroendocrine	I (T1, N0, M0)	Laparoscopic appendectomy
8	71	1.2	Neuroendocrine	I (T1, N0, M0)	Open ileocecectomy with ileostomy
9	53	1.1	Neuroendocrine	I (T1, N0, M0)	Laparoscopic appendectomy
10	47	1.5	Neuroendocrine	I (T1, N0, M0)	Laparoscopic appendectomy
11	47	0.7	Neuroendocrine	III (T4, N0, M0)	Laparoscopic appendectomy
12	46	1.1	Neuroendocrine	I (T1, N0, M0)	Laparoscopic appendectomy
13	40	0.9	Neuroendocrine	I (T1, N0, M0)	Open appendectomy
14	28	1.0	Neuroendocrine	I (T1, N0, M0)	Open appendectomy
15	26	1.1	Neuroendocrine	I (T1, N0, M0)	Open appendectomy
16	25	1.4	Neuroendocrine	I (T1, N0, M0)	Open appendectomy
17	23	1.1	Neuroendocrine	I (T2, N0, M0)	Open appendectomy
18	10	1.1	Neuroendocrine	I (T1, N0, M0)	Laparoscopic appendectomy
19	9	1.1	Neuroendocrine	I (T1, N0, M0)	Laparoscopic appendectomy
20	64	4.5	LAMN	–	Laparoscopic appendectomy
21	58	1.5	LAMN	–	Laparoscopic appendectomy
22	56	2.5	LAMN	–	Laparoscopic appendectomy
23	55	1.5	LAMN	–	Laparoscopic appendectomy
24	54	4.5	LAMN	–	Laparoscopic appendectomy converted to open right hemicolectomy
25	50	1.2	LAMN	–	Open right hemicolectomy with resection of terminal ileum and ileocolic anastomosis
26	47	5.5	LAMN	–	Laparoscopic appendectomy
27	42	2.1	LAMN	–	Open appendectomy

*LAMN* low-grade appendiceal mucinous neoplasm

**Fig. 3:**
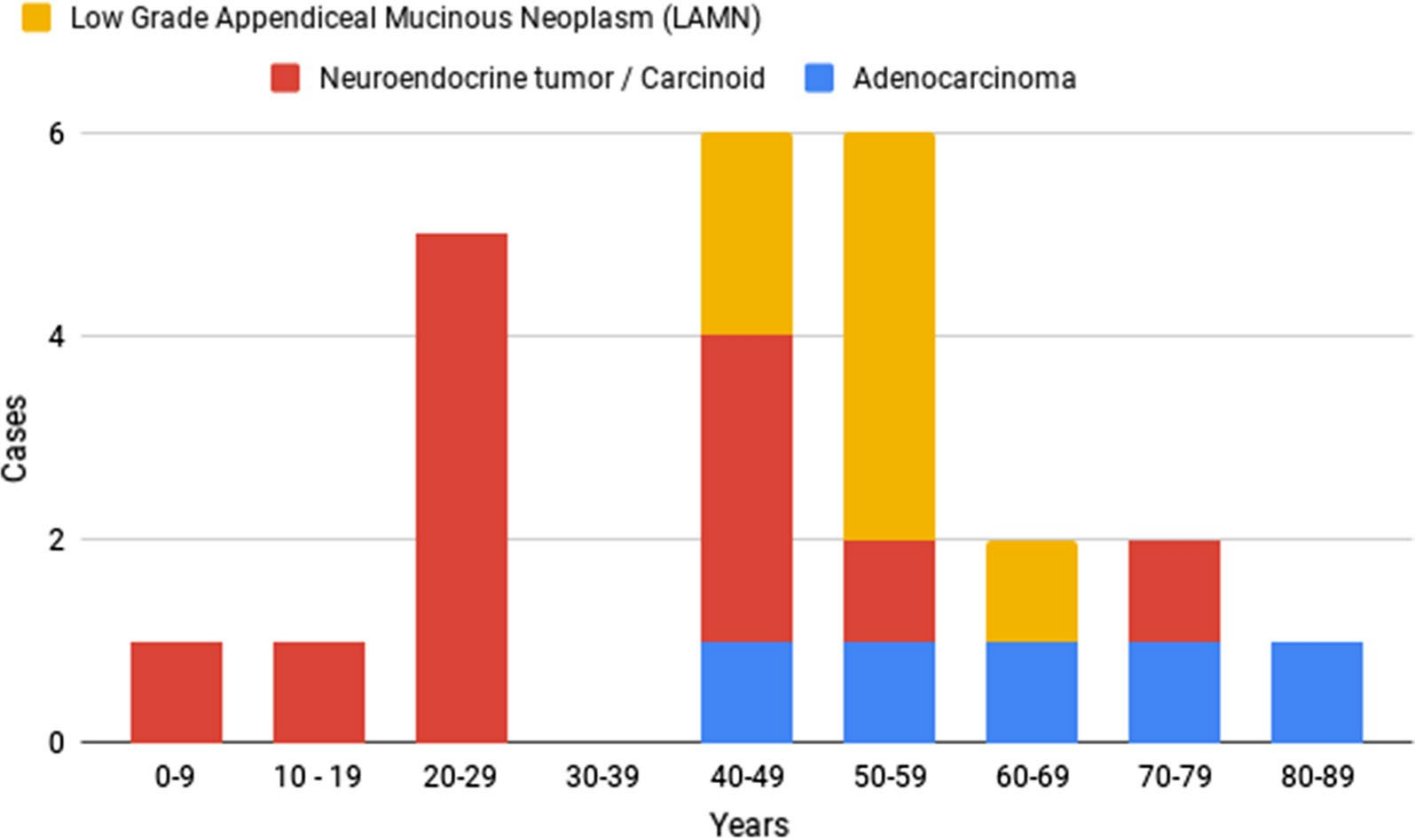
The incidence of adenocarcinoma, neuroendocrine, and low grade appendiceal mucinous neoplasm (LAMN) from two Bronx municipal hospitals for the period 2010 through 2018 categorized into 10-year age groups

A representation of patients divided by type of neoplasm and age is presented in Fig. 3. Adenocarcinoma only appeared in patients older than the age of 40 in the local cohort. Overall, older median age was associated with appendiceal adenocarcinoma: 65.0 IQR [52.8–75.3] *vs.* 47.0 IQR [25.5–54.5] years (p = 0.004; Table 1).

## Discussion

In this study we reviewed 2124 cases of acute appendicitis in two municipal hospitals. From our local experience, all cases of appendiceal adenocarcinoma were identified in patients older than 40 years, while cases of adenocarcinoma were reported in patients under 40 years in the NSQIP analysis. These data concur with a similar NSQIP analysis performed by Lu, et al. between 2016 and 2017. Furthermore, these results were in agreement with those established in prior reports that support a significant association between increased risk of malignancy and increasing age in patients who have undergone surgical appendectomy for presumed appendicitis [17]. The rate of appendectomy at Jacobi Medical Center and North Central Bronx Hospital for acute appendicitis was 94%, which also matched the United States NSQIP rate of 95%. The rate of appendiceal cancer at Jacobi and North Central Bronx Hospital of 1.4% was higher than the observed NSQIP rate of 0.53% for appendiceal cancer in acute appendicitis.

The incidence of appendiceal cancer rose from 2000 to 2009 in an analysis of the “Surveillance, Epidemiology, and End Results” (SEER) database [18]. In the setting of a progressively aging population, there is an observed increase in appendicitis in older adults. The management patterns of acute appendicitis are also evolving. Over the last several years, numerous studies have determined safety and efficacy of non-operative treatment with specific indications [1, 2, 7, 19–28]. Healthcare providers have been eager to adapt this new modality without consideration for possible long-term outcomes and oncologic ramification.

The COVID-19 pandemic has forced surgeons to alter their strategies in the management of acute appendicitis. Non-operative management, as well as the use of open appendectomy, reflect the poor guidelines available during the early stages of the SARS-CoV-2 pandemic [11]. This evolution has accelerated during the COVID-19 pandemic as non-operative management was encouraged by results from the CODA Trial [14] and American College of Surgeons guidelines [13].

The risk of appendiceal neoplasm increases with severe, complicated appendicitis [29]. Nevertheless, the severity of appendicitis may not be adequately determined by cross-sectional imaging [30]. CT failed to identify complicated disease in 187 of 837 patients (22%) thought to have uncomplicated disease based on imaging characteristics alone. Nevertheless, in patients with few or no risk factors for perforation, surgery can be safely postponed up to 7 h. These findings may have real clinical consequences if patients with complicated disease are not recognized and are offered non-operative management.

To date, the only gold standard confirmatory test for appendiceal cancer is appendectomy that is usually performed for the presentation of acute appendicitis [31–33]. In a recent prospective study of acute complex appendicitis, patients with complicated appendicitis who underwent either surveillance or interval appendectomy were found to have an exceedingly high rate of malignancy that even resulted in premature discontinuation of a prospective study [17, 29, 34, 35]. Our findings corroborate recent findings in prospectively collected data, analyzed post-hoc, from the MUSTANG trial [15, 16]. In our local study, 25 of 27 patients (93%) had an appendiceal diameter greater than 1 cm (Table 2). Taken together we concur that age older than 40 and an appendiceal diameter greater than 1 cm on CT imaging must be considered risk factors for malignancy when considering appendectomy at initial presentation or at a later date [15, 16, 36]. Patients with acute appendicitis managed with non-operative treatment should be followed with screening colonoscopy and interval, full-dosed contrast enhanced CT imaging based on recommendations from the World Society of Emergency Surgery [37].

Analysis of the NSQIP data demonstrated that the incidence of appendiceal neoplasm was higher in older adults, with a marked increase after 50 years. Nonetheless, this incidence was lower than that observed in our urban, Bronx-based population (0.53% vs. 1.4%). There are several explanations for this discrepancy. NSQIP does not capture every case of appendicitis. This leaves open the possibility that cases of appendiceal neoplasm were missed. This deficiency combined with the larger sample size may lead to a decrease in relative percentages of appendicitis in the NSQIP sample. Another point of possible discrepancy involved the pathologic diagnosis of appendiceal cancer. In the local group, anatomic pathology was performed and reviewed by one group. In NSQIP the data input was entered at the time of initial diagnosis based on a pathology report. It is possible that diagnostic criteria may vary from facility to facility across a national database. Regardless, the overall trends were similar. The incidence of appendiceal neoplasm, in particular appendiceal adenocarcinoma, increased with age and highlighted the need to consider the possibility of underlying malignancy in acute appendicitis in adults over the age of 40.

Interestingly, our analysis of NSQIP data in patients with appendiceal cancer show that cancer, and in particular appendiceal adenocarcinoma can also occur in patients younger than 50 years of age. Our local data also suggest that the incidence of adenocarcinoma directly correlates with age. However, our local study was limited by its retrospective design and review of patient data from a now unmaintained, older electronic medical record. In the emergency surgery setting, patient follow-up was poor. Our municipal hospital system also serves underrepresented and an economically disadvantaged patient population with limited adherence in all surgical settings. Furthermore, the practice patterns at our institution, with inclusion of earlier data, reflected a surgical practice pattern that may be limited at other centers, especially outside the United States.

Appendiceal neoplasms are an orphan disease with a heterogeneous entity that escape straightforward classification or management [38–40]. The “screening test” for appendiceal cancer is appendectomy for patients who present emergently with acute appendicitis. Appendiceal cancer is rarely found by colonoscopy or as an incidental finding on CT scan or abdominal surgery for other reasons [41]. However, delayed diagnosis for appendiceal adenocarcinoma may have significant changes in staging and outcome. Further study of these tumors in the acute setting is required to adequately screen patients who may not be eligible for medical management or whose atypical presentation may highlight the need for further oncologic workup prior to surgical intervention.

## Conclusions

Appendiceal neoplasms are a heterogeneous group of diseases that vary significantly in oncologic outcomes and are often diagnosed in the setting of acute appendicitis. Given the higher incidence of adenocarcinoma in older patients with appendicitis, patients over 40 years of age should be counseled regarding the risk of malignancy when considering non-operative therapy.

## Data Availability

De-identified patient data are available for external review upon reasonable request. Requests for identified patient data will be submitted to the Internal Review Board of Albert Einstein College of Medicine for consideration.
